# Characterisation of heart failure with normal ejection fraction in a tertiary hospital in Nigeria

**DOI:** 10.1186/1471-2261-9-52

**Published:** 2009-11-18

**Authors:** Adedeji K Adebayo, Adewole A Adebiyi, Olulola O Oladapo, Okechukwu S Ogah, Akinyemi Aje, Dike B Ojji, Ayodele O Falase

**Affiliations:** 1 Division of Cardiovascular Medicine, Department of Medicine, University College Hospital, Ibadan, Oyo State, Nigeria; 2 College of Medicine University of Ibadan, Ibadan, Nigeria

## Abstract

**Background:**

The study aimed to determine the frequency and characteristics of heart failure with normal EF in a native African population with heart failure.

**Methods:**

It was a hospital cohort study. Subjects were 177 consecutive individuals with heart failure and ninety apparently normal control subjects. All the subjects underwent transthoracic echocardiography. The group with heart failure was further subdivided into heart failure with normal EF (EF ≥ 50) (HFNEF) and heart failure with low EF(EF <50)(HFLEF).

**Results:**

The subjects with heart failure have a mean age of 52.3 ± 16.64 years vs 52.1 ± 11.84 years in the control subjects; p = 0.914. Other baseline characteristics except blood pressure parameters and height were comparable between the group with heart failure and the control subjects. The frequency of HFNEF was 39.5%. Compared with the HFLEF group, the HFNEF group have a smaller left ventricular diameter (in diastole and systole): (5.2 ± 1.22 cm vs 6.2 ± 1.39 cm; p < 0.0001 and 3.6 ± 1.24 cm vs 5.4 ± 1.35 cm;p < 0.0001) respectively, a higher relative wall thickness and deceleration time of the early mitral inflow velocity: (0.4 ± 0.12 vs 0.3 ± 0.14 p < 0.0001 and 149.6 ± 72.35 vs 110.9 ± 63.40 p = 0.001) respectively.

The two groups with heart failure differed significantly from the control subjects in virtually all echocardiographic measurements except aortic root diameter, LV posterior wall thickness(HFLEF), and late mitral inflow velocity(HFNEF). HFNEF accounted for 70(39.5%) of cases of heart failure in this study.

Hypertension is the underlying cardiovascular disease in 134(75.7%) of the combined heart failure population, 58 (82.9%) of the subjects with HFNEF group and 76(71%) of the HFLEF group. Females accounted for 44 (62.9%) of the subjects with HFNEF against 42(39.3%) in the HFLEF group (p = 0.002).

**Conclusion:**

The frequency of heart failure with normal EF in this native African cohort with heart failure is comparable with the frequency in other populations. These groups of patients are more likely female, hypertensive with concentric pattern of left ventricular hypertrophy.

## Background

Congestive heart failure has been recognized from ancient times [1]. The fact that heart failure remains a big problem today is not surprising considering that it is the final pathway of myriad of diseases that affect the heart[2-4]. Heart failure constitutes a pandemic which has not spared any group of people. In the western world where there are reliable epidemiological studies, the problem is grave indeed, not only in terms of the high morbidity and mortality, but also in terms of total cost to the different nations[5-7]. Even in the developing countries where not much data are available, it is known that what is lacking in ischemic heart disease- an almost exclusive preserve of the developed world, is made up for in hypertensive, diabetic and infective heart diseases leading to heart failure[5,8-11]

It is recognised that there is a group of patients presenting with the syndrome of congestive cardiac failure in the setting of a normal ejection fraction[12,13]. The syndrome in this group of patients known as diastolic heart failure is believed to be due to impaired myocardial relaxation and filling. There is therefore compensatory elevation of ventricular filling pressures to maintain normal forward cardiac output. The elevated left atrial pressure is transmitted to the pulmonary vascular bed resulting in pulmonary venous hypertension and its clinical manifestations [12,14].

Although this entity has been challenged on the basis of the presentation and symptomatology which is at least similar to the features of other conditions common in the elderly and, cardiovascular changes associated with aging [15,16], studies have shown definite abnormalities in diastolic function in patients fulfilling clinical criteria for heart failure in the setting of preserved ejection fraction [17-19]. This form of heart failure is also referred to as heart failure with normal ejection fraction or heart failure with preserved ejection fraction.

From previous studies, it is known that patients with heart failure and normal ejection fraction are typically elderly, often females, usually obese and frequently have hypertension and diabetes [4]. This study aimed to determine the pattern and the characteristics of heart failure with preserved ejection fraction in subjects presenting to a tertiary institution in Ibadan, Nigeria.

## Methods

### Study location

The study was carried out at the cardiology unit of the department of medicine of the University College Hospital, Ibadan, Nigeria. The University College Hospital Ibadan is the only tertiary health care facility located in the city of Ibadan in south western Nigeria with more than three million inhabitants.

### Study population

Eligible patients were adult men and women aged 18 years and above, presenting with syndrome of heart failure. A total of 205 consecutive subjects with heart failure were screened. 177 subjects were found eligible after twenty one subjects were excluded on account of suboptimal echocardiographic image qualities and seven subjects on account of co-existent chronic obstructive airway disease based on history or clinical examination or chest radiograph. A second group consisted of ninety apparently normal men and women who had no clinical history of cardiovascular or any other medical condition. They were recruited from the medical (27 subjects, constituting 30%) and non-medical staff of the University College Hospital Ibadan and patients' relations. The two groups were comparable in their age and sex ratios.

Informed consent was obtained from all the study participants before enrolment into the study. Ethical clearance was obtained from the joint University of Ibadan and University College Hospital ethical committee.

### Clinical evaluation

Diagnosis of congestive heart failure was based on the Framingham's criteria for diagnosis of heart failure[20]. Major criteria were paroxysmal nocturnal dyspnoea, orthopnoea, raised jugular venous pulse, lung crepitations, cardiomegaly and gallop sounds. Minor criteria were ankle oedema, night cough, dyspnoea on exertion, hepatomegaly, pleural effusion and tachycardia.

Subjects were weighted without shoes and in light clothing on a standard beam balance. Height was measured to the nearest centimetre using anthropometrical plane with subjects not putting on shoes or headgear.

Body mass index (BMI) was calculated using the formula BMI = Weight (kg)/(height)^2 ^Body surface area was calculated using the formula of Dubois[21].

Diagnosis of heart failure with normal ejection fraction required:

(1) Minimum of two major criteria or one major criterion plus two minor criteria.

(2) Left ventricular ejection fraction (LVEF) equal to or more than 50%.

Heart failure with reduced/low ejection fraction required (1) above and LVEF < 50%.

Hypertension was deemed present if systolic BP ≥ 140 mmHg and or diastolic BP ≥ 90 mmHg[22,23] on at least 2 occasions or if patient was receiving anti-hypertensive drug treatment. Diabetes mellitus was diagnosed based on symptoms with Random blood sugar >200 mg/dl, fasting blood sugar (FBS) >126 mg/dl (R) or subjects on hypoglycaemic medications[24].

The BP was recorded in the right arm of subjects who have rested for at least 5 minutes with an appropriately sized cuff.

Coronary artery disease was considered present and a cause of heart failure when there was evidence of definite myocardial infarction from history, ECG or echocardiogram or if there is a long history of stable angina pectoris.

Valvular heart disease was considered the primary cause of heart failure when there was echocardiographic evidence of valvular heart disease of more than moderate severity.

Dilated cardiomyopathy was diagnosed based on dilated cardiac chambers with increased left ventricular mass and normal wall thickness

Echocardiogram was also employed to exclude pericardial and congenital heart diseases.

### Echocardiography

Echocardiography assessment was carried out with ALOKA SSD - 1700 echo machine which was equipped with a 3.5 MHz linear array transducer. Two dimensional guided M-mode echocardiography was performed on each subject in the left lateral decubitus position. All measurements were made according to the American Society of Echocardiography leading edge to leading edge criteria[25]Average of measurements in three cardiac cycles were taken with simultaneous ECG recording.

LV internal diameter and interventricular septal and posterior wall thickness was measured at end diastole and end systole. Left ventricular (LV) systolic performance was assessed using the fractional shortening of the left ventricle and the ejection fraction.

LV ejection fraction was calculated using the Teichholz calculation formula[26].

Fractional shortening was calculated from LV internal dimensions in diastole and systole

Left ventricular mass(LVM) was calculated using the Devereux-modified ASE cube formular[27].

Where, VSTd is the ventricular septal thickness in diastole; PWTd is the posterior ventricular wall thickness in diastole and LVIDd is the left ventricular internal diameter in diastole

Relative wall thickness(RWT) was calculated using[28].

The aortic root was measured at end diastole and the left atrium was measured at end-ventricular systole, including the thickness of the posterior wall of the aorta

Left ventricular diastolic function was evaluated by studying the filling dynamics of the left ventricle. The mitral inflow velocities were measured from the apical four chamber view with pulsed-wave Doppler and with the sample volume positioned at the tip of the mitral valve leaflets. This inflow characteristics were quantified by measuring the transmitral "E" wave velocity (peak early mitral inflow velocity) and the "A" wave velocity (peak atrial mitral inflow velocity), the E/A ratio and the deceleration time (DT):(time interval of Peak E wave velocity to its extrapolation to the baseline) [29-31]. Two experienced physicians blinded to the clinical data of the patients carried out the echocardiographic studies. Intraobserver concordance correlation coefficient varied between 0.76 to 0.98 while interobserval concordance varied between 0.82 to 0.96 in our laboratory.

### Data management and statistical analysis

Statistical analysis was performed using SPSS software version 10.0(SPSS, Inc. Chicago Illinois). Means were expressed as means (standard deviation) while proportions were expressed as count (percentages). Comparison of categorical variables between the groups was by the chi square test, while continuous variables were compared using the students't' test for independent groups. Analysis of variance was used to comparison of mean between 3 or more groups. Non parametric tests were used where appropriate. A two tailed p value < 0.05 was considered to be significant.

## Results

Table 1 shows the baseline characteristics of all the 267 adult Nigerians comprising 177 subjects (patients with clinical features of congestive cardiac failure) and 90 (apparently healthy) subjects in the control group, who were recruited into the study. There was no significant difference in the age, gender distribution or weight but the control subjects were taller. The systolic blood pressure and mean arterial pressure were higher in the subjects with heart failure but the other blood pressure parameters were comparable.

**Table 1:** Baseline characteristics of the Subjects

Variables	Heart failure N = 177	Controls N = 90	P value
Age	52.3(16.64)	52.1(11.84)	0.914
Gender(m/f)	91/86	42/48	0.610
Weight	67.4(16.11)	69.2(14.55)	0.401
Height	162.3(10.73)	164.9(8.22)	0.040
BMI	26.9(4.55)	25.5(5.31)	0.545
SBP	125.4(23.53)	119.6(8.49)	0.004
DBP	79.6(16.06)	77.1(7.72)	0.090
PP	45.5(20.48)	42.4(7.37)	0.073
MAP	94.3(17.71)	91.3(7.20)	0.047

BMI = Body mass index, SBP = Systolic blood pressure, DBP = Diastolic blood pressure, PP = Pulse pressure, MAP = Mean arterial blood pressure.

Stratifying the subjects with heart failure into 2 groups based on whether the ejection fraction is preserved or reduced and comparing with control subjects (table 2), reveals a comparable mean age between the three groups. The group with normal ejection fraction had more females, a higher systolic blood pressure, pulse pressure and body mass index compared with both the subjects with reduced ejection fraction and the control subjects. The diastolic blood pressure and mean arterial pressure are comparable among the three groups. Heart failure with normal ejection fraction accounted for 39.5% of the subjects with heart failure with the rest accounted for by heart failure with reduced ejection fraction.

**Table 2:** Analysis of variance of the 2 groups of subjects with heart failure and the control subjects

Variables	HFLEF(A) N = 107	HFNEF(B) N = 70	Controls(C) N = 90	P value	P value A vs B	P value A vs C	P value B vs C
Age	51.1(15.78)	54.2(17.83)	52.1(11.84)	0.385	0.540	0.931	0.788
Gender	65/42	25/44	48/42	0.010	-	-	-
Weight	65.2(12.09)	70.8(20.39)	69.2(14.55)	0.051	0.121	0.125	0.918
Height	164.0(8.20)	159.6(13.31)	164.9(8.22)	0.003	0.043	0.833	0.012
BMI	24.2(4.00)	30.9(4.152)	25.5(5.31)	0.004	0.001	0.886	0.090
SBP	121.0(22.29)	132.2(23.89)	119.6(8.49)	<0.0001	<0.0001	0.910	<0.0001
DBP	80.2(17.17)	78.8(14.30)	77.1(7.72)	0.274	0.918	0.283	0.767
PP	40.4(19.23)	53.4(19.98)	42.4(7.37)	<0.0001	<0.0001	0.685	<0.0001
MAP	92.9(19.03)	96.6(15.43)	91.3(7.20)	0.041	0.404	0.806	0.028

HFNEF = Heart failure with normal ejection fraction, HFLEF = Heart failure with low ejection, BMI = Body mass index, SBP = Systolic blood pressure, DBP = Diastolic blood pressure, PP = Pulse pressure, MAP = Mean arterial blood pressure.

Hypertension was the most frequent underlying cardiovascular disease across all the heart failure groups. Hypertension was present in 75.7% of the combined heart failure group, 71% of the group with heart failure and reduced EF and 82.9% of the group with heart failure with normal EF.

Dilated cardiomyopathy accounted for 13.6% of the underlying cardiovascular factors. Valvular heart disease, mainly rheumatic was found in 7.9% of the heart failure subjects. Ischaemic and restrictive heart disease accounted for 1.7% and 0.6% of the cases respectively. The only case of restrictive heart disease was histologically confirmed to be due to amyloidosis. Eleven subjects were found to have Diabetes mellitus. All the subjects with diabetes also had hypertension.

Table 3 shows the analysis of variance of the echocardiographic parameters of the subjects with heart failure and the controls. The 2 groups with heart failure have comparable left atrial size, septal and posterior wall dimensions but a trend towards a higher indexed left ventricular mass in the group with reduced ejection fraction. The group with reduced ejection fraction had a wider LV dimension both in systole and diastole and a lower relative wall thickness. Apart from aortic root dimension(both groups with heart failure), posterior wall thickness in diastole(group with reduced EF), relative wall thickness(group with normal EF) which are comparable with the control group, the heart failure group differed significantly from the control group in measure of left ventricular geometry.

**Table 3:** Echocardiographic parameters of the subjects

Variables	HFNEF(A) n = 70	HFLEF(B) n = 107	Controls(C) n = 90	ANOVA P-value	P value A vs B	P value A vs C	P value B vs C
LA(cm)	4.2(1.03)	4.8(1.0.99)	3.1(0.43)	<0.0001	0.540	<0.0001	<0.0001
AORTA(cm)	2.8(0.82)	2.8(0.604)	2.8(0.38)	0.403	0.858	0.966	0.936
IVSTd(cm)	1.09(0.277)	1.04(0.301)	0.88(0.14)	<0.0001	0.486	<0.0001	<0.0001
PWTd(cm)	0.95(0.21)	0.91(0.27)	0.87(0.13)	0.0413	0.531	0.025	0.578
LVIDd(cm)	5.2(1.22)	6.2(1.39)	4.5(0.54)	<0.0001	<0.0001	<0.0001	<0.0001
LVIDs(cm)	3.6(1.24)	5.4(1.35)	2.9(0.48)	<0.0001	<0.0001	<0.0001	<0.0001
RWT	0.4(0.12)	0.3(0.14)	0.4(0.07)	<0.0001	<0.0001	0.777	<0.0001
LVM/HT^2.7^(g/^m2.7^)	50.8(20.93)	55.6(21.6)	29.6(10.08)	<0.0001	0.063	<0.0001	<0.0001
FS (%)	31.6(10.35)	12.5(4.74)	35.9(7.29)	<0001	<0.0001	0.012	<0.0001
EF (%)	65.5(11.22)	32.0(11.03)	72.7(8.57)	<0.0001	<0.0001	<0.0001	<0.0001
E(m/s)	0.7(0.39)	1.1(0.72)	0.6(0.16)	<0.0001	0.964	0.028	0.006
A(m/s)	0.5(0.25)	0.4(0.24)	0.5(0.14)	0.021	0.081	0.985	0.002
E/A	1.7(1.01)	2.1(0.91)	1.3(0.39)	<0.0001	0.117	0.003	<0.0001
DT(ms)	149.6(72.35)	110.9(63.40)	178.6(41.13)	<0.0001	0.001	0.011	<0.0001

HFNEF = Heart failure with normal ejection fraction, HFLEF = Heart failure with low ejection, LA = Left atrium, IVSd = Interventricular septal wall thickness in diastole, PWTd = Posterior wall thickness in diastole, LVIDd = Left ventricular internal diameter in diastole, RWT = Relative wall thickness, LVM = Left ventricular mass, HT = Height, FS = Fractional shortening, EF = Ejection fraction, E = Early filling velocity, A = Late filling velocity, DT = Deceleration time

The group with heart failure and reduced EF had a reduced deceleration time of early transmitral flow velocity (E). Otherwise measures of transmitral flow are comparable between the 2 groups. Compared with the control subjects, both groups have higher early mitral inflow velocity (E) and higher ratio of early inflow velocity to the atrial mitral inflow velocity (E/A ratio) and a lower deceleration time of early transmitral flow velocity (E). Additionally, the group with reduced EF have a lower late transmitral flow velocity (A).

## Discussion

The study showed that patients presenting in heart failure are more likely to have a higher left ventricular mass, thicker septum and more dilatation of the left atrium and the left ventricle when compared with normal subjects. It also showed that subjects with heart failure and preserved ejection fraction and subjects with reduced EF have comparable left ventricular wall thickness and left atrial dilatation. The major differences between these 2 groups are more LV dilatation and reduced deceleration time of E in the subjects with reduced EF. Heart failure with normal ejection fraction occurred in one out of every three heart failure patients. Hypertension was the most frequent underlying cardiovascular disease associated with heart failure and also heart failure with preserved ejection fraction in this African population of subjects with heart failure.

In the study the mean age of patients with heart failure was 52.3 ± 16.6 years which is comparable with the findings in previous studies by Falase et al[9] over 2 decades ago and recently by Oyati et al[32] in similar racial groups. The age is higher in Caucasian studies. Senni et al [20] reported mean age of 77.3 ± 12.1 years and Cowie et al[3] reported a median age of 76-78 years for women and men in studies among Caucasian subjects. This trend is consistent for studies in Caucasian populations. Agoston et al [33] comparing black and white subjects with heart failure found that blacks and whites with preserved ejection fraction had comparable age while blacks with reduced ejection fraction were younger. The differences found in the studies are most likely due to a combination of differences in race: white vs black and environmental influences since Africans in diaspora did not show exactly same characteristics with the native Africans in this study.

The mean age of the group presenting with heart failure and normal ejection fraction and the group presenting with reduced ejection fraction was not significantly different. This finding is similar to previous studies in Caucasian populations by Varela-Roman et al[34] and Senni et al[20] who compared patients with normal EF with those who had reduced EF. There was a female predominance in the group with preserved EF. Females accounted for 62.9% of the subjects with normal EF against 39.3% in the group with reduced EF (p = 0.002). Female preponderance in subsets with heart failure and normal EF has been consistent in the few studies that have attempted to determine gender differences. For instance Vasan et al[2] reported that 65% of patients who presented with normal ejection fraction were women in a case control study. Heart failure with normal ejection fraction was found in 39.5% of the patients who presented in heart failure in our study. This figure is comparable with the figure of 40-71% quoted in previous studies in other populations[35].

The systolic blood pressure and pulse pressure were significantly different between the group with normal ejection fraction and the group with reduced ejection fraction. These parameters were higher in the former. These findings are consistent with the fact that hypertension was found to be a more frequent aetiology of heart failure in the group with normal ejection fraction than in the group with reduced ejection fraction. Also, a narrow pulse pressure is an expected accompaniment of the low ejection fraction characterising heart failure with low EF. Ahmed et al [36] had demonstrated a distinct relationship between higher systolic blood pressure and heart failure with preserved ejection fraction.

### Left ventricular geometry

Comparing the heart failure groups, it was found that the end diastolic diameter, end systolic diameters were significantly larger in the group with low ejection fraction, while expectedly the relative wall thickness was higher in the group with preserved ejection fraction. Distinct relationship between left ventricular dilatation and poor systolic function is well established. Devereux et al[37] and Tsutsui et al[38] also documented that the patients with reduced ejection fraction tended to have relatively thinner walls and more dilated ventricles when compared with the group with normal ejection fraction that tended to have a more concentric hypertrophy pattern.

### Diastolic dysfunction

The shorter deceleration time(DT) in the group with heart failure and reduced ejection fraction is similar to the findings by Devereux et al[37]. This is likely due to the expected natural history of the failing heart in which at the later stages of severe heart failure there is increasing restrictive left ventricular filling. The higher mitral E velocity and E/A ratio in the two groups with heart failure when compared with the control subjects and the lower transmitral A velocity demonstrable only in the group with low ejection fraction is in support of the postulation that heart failure occur as a spectrum of abnormalities beginning from diastolic dysfunction and only mild systolic dysfunction detectable only by more sensitive modalities, progressing to the extreme of severe systolic and diastolic dysfunction. The finding by Kitzman et al[39], of no significant difference between the mitral inflow characteristics of the group with heart failure and normal EF and the group with reduced EF, further strengthens the position that the diastolic dysfunction may also be mild initially. It is very likely that more sensitive modalities or multiple modalities for assessing diastolic dysfunction would have demonstrated at least some degree of diastolic abnormalities in these patients. The body mass index was higher in the subset of patients with heart failure and normal EF. This is consistent with findings from previous studies[4]. The relationship between obesity and hypertension and diastolic dysfunction may be responsible for this finding.

### Aetiology of heart failure

Hypertension was the overall leading cause of heart failure in this study. Furthermore hypertension was implicated even more frequently in patients with preserved ejection fraction. This is consistent with the findings in some other populations [40,41]. The implication is that although hypertension is the commonest cause of heart failure among black Africans[42,9], its definite relationship with heart failure with normal ejection fraction is obvious still. The spectrum of underlying cardiovascular diseases in this study when compared with a previous study by Senni et al[20], showed a similarity in the range of aetiological factors for heart failure as a whole, except for the prominence of ischaemic heart disease and the absence of valvular heart disease in their study carried out in a predominantly Caucasian population. When they compared the underlying cardiovascular disease in the group with normal ejection fraction and the group with reduced ejection fraction, hypertension was the most frequent factor in the two groups. This trend is similar to the findings in this study. The difference is that hypertension may be a much more important underlying factor for heart failure in native African populations.

Although there is considerable debate whether HFNEF can evolve into HFLEF, we are of the view that in Africa where hypertension is the main aetiology, this may be the case. Some studies have suggested this in other populations and there is need to study the natural history HFNEF in native Africans [43-45].

### Limitation of the study

The study, a hospital based, carried out in a single centre might not be completely representative of the findings in the community or in other centres, hence a multicentred and/or community based study would be desirable to fully characterise heart failure with preserved EF in this population group.

Use of other modalities of assessing diastolic function such as pulmonary venous flow velocities, tissue Doppler echocardiography or invasive measurements could have helped to further establish and characterise diastolic dysfunction in this study population. These were not included in the study protocol.

We also recognise the use of Teicholtz formula rather than the modified Simpson's method in the assessment of the ejection fraction (in the presence of ventricular dilatation) as a limitation of the study

## Conclusion

Heart failure with normal ejection fraction constitutes a significant part of the heart failure burden in this native African cohort and the frequency of 39.5% found in this study is comparable with findings in other populations. Hypertension was the most common underlying cardiovascular condition overall and in the different heart failure groups, the highest relative frequency being in the group with heart failure and normal ejection fraction. Patients with heart failure and normal ejection fraction are more likely female with higher body mass index, wider pulse pressure, thicker septum, and a more concentric pattern of hypertrophy when compared with the group with reduced ejection fraction.

## Competing interests

The authors declare that they have no competing interests.

## Authors' contributions

AKA, OOO, AAA, AOF participated in the original design of the study. OSO, AA, and DBO participated in the collection of data. AKA and OSO drafted the manuscript. All authors contributed in the interpretation of the study data and have seen and approved the final version of the report.

## Pre-publication history

The pre-publication history for this paper can be accessed here:

http://www.biomedcentral.com/1471-2261/9/52/prepub
